# A randomized clinical trial on the effect of a lidocaine patch on shoulder pain relief in laparoscopic cholecystectomy

**DOI:** 10.1038/s41598-020-80289-y

**Published:** 2021-01-13

**Authors:** Ha Yeon Kim, Jong Bum Choi, Sang Kee Min, Min Ying Chang, Gang Mee Lim, Ji Eun Kim

**Affiliations:** Department of Anesthesiology and Pain Medicine, Ajou University School of Medicine, 164, World cup-ro, Yeongtong-gu, Suwon, Republic of Korea

**Keywords:** Gastroenterology, Health care, Medical research, Signs and symptoms

## Abstract

The incidence of laparoscopy-related shoulder pain reaches 90% in women. We evaluated the effect of lidocaine patch 5% on the shoulder pain after laparoscopic cholecystectomy (LC) in female patients. Total 63 female patients were randomly allocated to patch group (n = 31) and control group (n = 32). Patch group received lidocaine patch 5% and dressing retention tape on both shoulder, and control group received only dressing retention tape. Abdominal and shoulder pains were evaluated with rating on numeric rating scale (0 = no pain and 10 = the worst pain) at baseline and at 30 min, 6 h, 24 h, and 48 h after surgery. There were no significant differences in patient characteristics and operation details. The overall incidence of shoulder pain was significantly lower in patch group than in control group (42% vs. 78%, P = 0.005). The severity of shoulder pain also was significantly reduced in patch group compared to control group at 24 h and 48 h after surgery (P = 0.01 and P = 0.015, respectively). Complications related to lidocaine patch were not found except nausea. Lidocaine patch 5% reduced the incidence and severity of postoperative shoulder pain in female patients undergoing LC without complications.

## Introduction

Laparoscopic cholecystectomy (LC) has become a standard treatment for gall bladder disease because of advantages such as smaller incision, shorter hospital stays and faster recovery compared with open cholecystectomy^1^. Although LC is considered as a less painful procedure, patients may experience shoulder pain after undergoing LC. Shoulder pain after surgery occurs rarely in open surgery, but its incidence rises to 30–60% in general laparoscopic surgery, reaching 90% in women^2–4^. Some patients unexpectedly may experience severe pain in laparoscopic surgery than in aggressive, major surgeries^4,5^. However, laparoscopy-related shoulder pain is poorly responsive to analgesics^4^. Therefore, the efforts to prevent the laparoscopy-related shoulder pain are essential.

Although the mechanism has not been fully clarified, laparoscopy-related shoulder pain is generally considered to develop due to diaphragmatic irritations from direct injury, stretching, or CO_2_ gas^2,3,6^. Clinically, diaphragmatic irritation manifests as referred pain in the shoulder arising from the phrenic nerve^4,7^. Interventions to reduce shoulder pain after LC aim to minimize diaphragmatic irritation through low-pressure pneumoperitoneum^8^, intraperitoneal instillation of analgesics^9^, drain suction^10^, active gas aspiration^11^ or phrenic nerve block^12^. However, local anesthesia applied to the area of referred pain, and not initial area, has also been shown to be effective in reducing referred pain in the tibialis muscle^13^; further, trigger point injection or a eutectic mixture of local anesthetics (EMLA) cream applied to the shoulders, not the diaphragm, significantly reduced shoulder pain after laparoscopic hysterectomy^14^.

Lidocaine patch 5% is a topical analgesic that interrupts pain signals in peripheral nociceptors with minimal systemic absorption and few adverse effects^15^. In a randomized controlled study of myofascial pain syndrome, lidocaine patch 5% decreased the symptoms of pain and the sensation of the skin as effectively as trigger point injection^16^. We hypothesized that application of lidocaine patch 5% to the shoulder could also reduce the severity of shoulder pain after LC.

The aim of this study was to evaluate the analgesic effect of lidocaine patch 5% on shoulder pain after LC in female patients.

## Methods

This randomized, double-blinded, prospective, parallel-group study was conducted with patients undergoing LC at the Ajou University Health System between February 2017 and September 2017. The Ajou Hospital Institutional Review Board affiliated to Ajou University School of Medicine (protocol number: AJIRB-MED-CT4-16-076) approved the study protocol (ClinicalTrial.gov, NCT02827136, 11/07/2016). This study was conducted in proportion to relevant guidelines and regulations. After obtaining written informed consent from all participants, female patients with American Society of Anesthesiologists (ASA) physical status I, II or III aged 19–85 years, were included. LC included both elective and emergence surgeries performed in the day time (8:00–17:00). Exclusion criteria were as follows: histories of trauma, infection, surgery, or chronic pain involving the shoulders, hypersensitivity to local anesthetics, chronic abuse of opioids, impaired liver or renal dysfunction, or denial to participate in this study.

### Interventions

Participants (n = 64) were randomized to one of two groups by randomization generator (http://www.random.org) at 1:1 ratio by J.E.K.: the patch group (n = 32) and the control group (n = 32). Assigned group was concealed in a sealed, opaque envelope. Immediately before anesthesia induction, the envelope was opened by an independent investigator who performed all interventions but was not participated in outcome assessment. The anesthesia provider, patients, and preoperative and postoperative outcome assessors did not know the assigned group throughout the study period.

None of the patients received premedication. On arrival to the operating room, basic monitoring including pulse oximetry, electrocardiography, and non-invasive blood pressure measurement was performed. Before anesthesia induction, lidocaine patches (10 × 14 cm; Lidotop, Teikoku Seiyaku Co., Kagawa, Japan) were applied to both shoulders of patients in the patch group; then, the lidocaine patches were covered with dressing retention tape (12 × 15 cm; Hypafix, BSN Medical GmbH, Hamburg, Germany). In the control group, only dressing retention tape (12 × 15 cm; Hypafix) was applied, also to both shoulders. The patients’ shoulders were covered with clothes; thus, the outcome assessors could not see it. For anesthesia induction, intravenous (IV) propofol 2 mg/kg and remifentanil 0.3 μg/kg were started and rocuronium 0.8 mg/kg was followed. After endotracheal intubation, mechanical ventilation was initiated. For maintenance of anesthesia, remifentanil was infused at a rate of 0.05–0.10 μg/kg/min, and sevoflurane 2–2.5% was used within a range of bispectral index score 40–60. In case of mean arterial pressure (MAP) < 60 mmHg or heart rate (HR) < 40 beats/min, IV ephedrine 4 mg or atropine 0.5 mg was administered, respectively. Approximately 10 min prior to the end of surgery, IV propacetamol 1 g was administered for postoperative analgesia. At the end of surgery, sevoflurane were discontinued, and the fresh gas flow was increased to 5 L/min. To reverse residual neuromuscular blockade, IV neostigmine 50 μg/kg plus glycopyrrolate 10 μg/kg were injected after confirming the train-of-four count > 2 using a nerve stimulator. After confirming adequate tidal volume, patients were extubated with maintaining the remifentanil infusion of 0.05 μg/kg/min to prevent the emergence cough. Then, the patients were transferred to a post-anesthesia care unit (PACU).

All procedures were carried out by two skilled surgeons with same method. LC were performed through three abdominal ports (10-mm infraumbilical camera, 5-mm subxipoid, and 5-mm right lateral subcostal ports). CO_2_ gas was inflated through infraumbilical Veress needle. Abdominal insufflation pressure was set at 12 mmHg. Drain was not inserted, and local anesthetics were not injected at peritoneum or port site.

### Data collection

The primary outcome of this study was the severity of shoulder pain after surgery. Preoperative variables included demographics, ASA physical status, and diagnosis. Intraoperative variables included anesthesia time, operation time, and amounts of crystalloid and bleeding. Hemodynamic data such as HR and MAP were collected at five time points: at baseline, at pneumoperitoneum, at 20 min and 30 min after pneumoperitoneum, and at the end of surgery. Pain included the abdominal pain, and overall, right, and left shoulder pains. The incidence of shoulder pain was evaluated based on the overall value of shoulder pain and defined as the number of patients who had a pain score that was higher than the value at baseline. “> abdominal pain” was defined as the number of patients who had worse shoulder pain compared with abdominal pain during the 48 h following surgery. “Alleviated pain” was defined as the number of patients who had less shoulder pain compared to value at baseline. The severity of pain was quantified on a numeric rating scale (NRS) ranging from 0 to 10 (0 = no pain and 10 = the worst pain) at five time points: at baseline, and at 30 min, 6 h, 24 h, and 48 h after surgery. The pain score at 48 h after surgery was investigated by phone call with the patient. Nausea was graded into four (1 = none, 2 = mild, 3 = moderate, and 4 = severe). IV ramosetron 0.3 mg was administered to with vomiting or nausea grade ≥ 3 or 4. The lidocaine patches and/or dressing retention tape were removed by the ward’s attending nurse at 12 h following surgery. Complications related to lidocaine patch 5% (skin erythema, pruritus, blisters, contact hypersensitivity, nausea, headache, and arrhythmia) were evaluated by the ward’s attending nurse at ward until discharge from the hospital.

### Postoperative pain treatment

On arrival to the PACU, IV fentanyl 1 μg/kg was administered as a rescue analgesic in patients reporting an NRS ≥ 5. At the ward, IV ketorolac 30 mg was administered at 8 h intervals on the day of surgery. In addition, IV nefopam 20 mg was administered as a rescue analgesic in patients reporting an NRS ≥ 5. At the postoperative day 1, the patient discharged with prescription drug, which was consist of oral acetaminophen/tramadol 325/37.5 mg at three times a day.

### Statistical analysis

To calculate the sample size, we focused on the severity of shoulder pain after surgery. In a previous study, the pain score of shoulder pain after LC was 4.43 ± 1.4^17^. Considering that a mean difference of 1.2 in pain score was significant^18^, 29 participants were required in each group for a type I error of 5% and a power of 90%. Considering a 10% dropout rate, a total of 64 patients (32 per group) were required.

Data are shown as mean ± standard deviation (or standard error), median (interquartile range), or number of patients (proportion). Normality of distribution was assessed with the Kolmogorov–Smirnov test. Parametric and nonparametric data were analyzed using Student’s t-test and the Mann–Whitney test, respectively. Categorical data were analyzed using the chi-square test or Fisher’s exact test. Repeated measured data were analyzed using the linear mixed model. When the interaction was statistically significant, the adjusted P value was obtained with Bonferroni correction. P < 0.05 was considered statistically significant. SPSS for Windows (version 25.0, SPSS Inc., Chicago, IL, USA) was used for statistical analyses.

## Results

**Figure 1:**
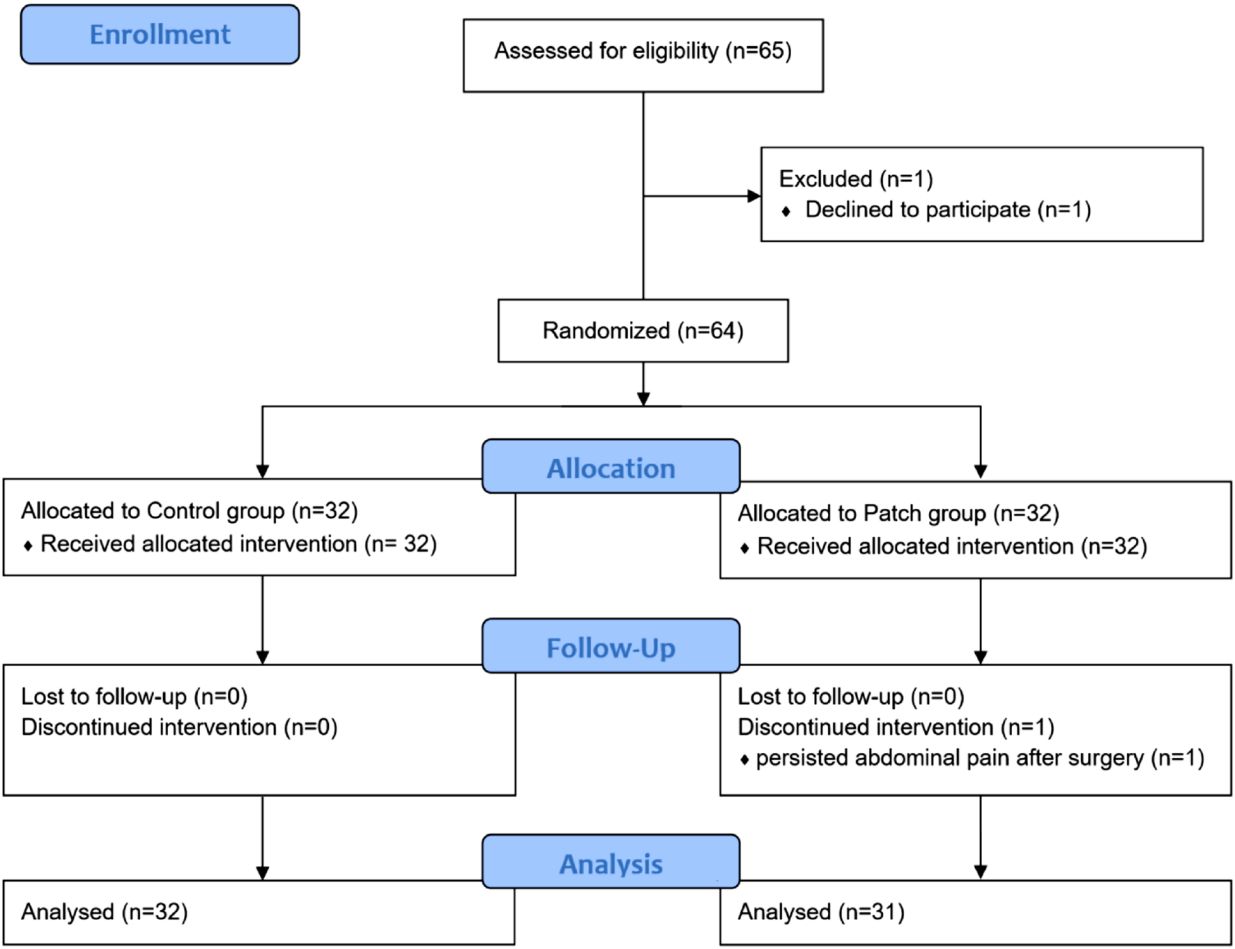
Flow diagram.

**Figure 2:**
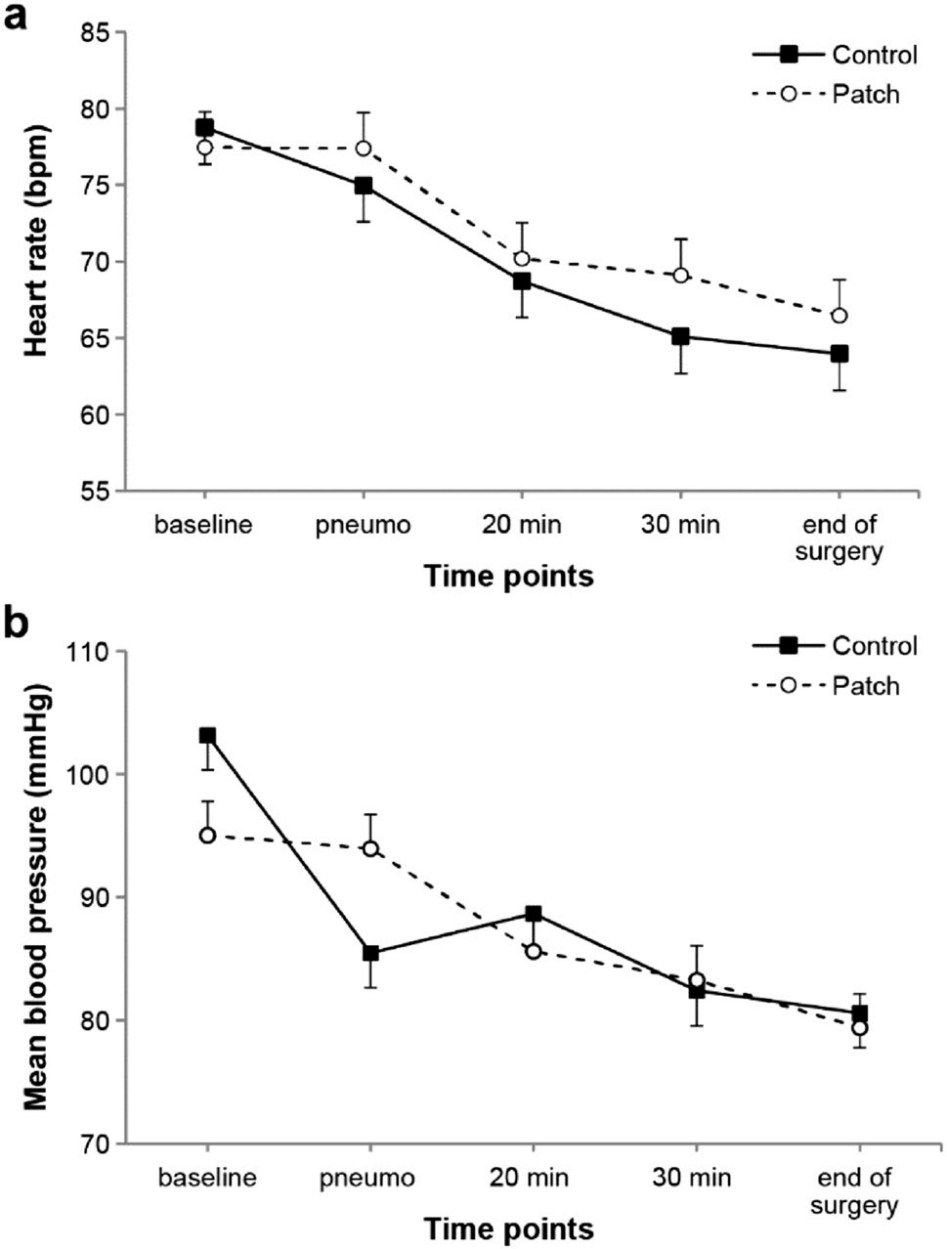
Changes of heart rate (**a**) and mean blood pressure during surgery (**b**). Values were expressed as mean ± standard error. *Baseline* before anesthesia induction, *pneumo* at pneumoperitoneum, *20 min* 20 min after pneumoperitoneum, *30 min* 30 min after pneumoperitoneum, *end of surgery* 10 min before the end of surgery.

Of the 64 patients included in this study between February 2017 and September 2017, one patient in the patch group dropped out due to persisting intolerable abdominal pain; finally, the data of 63 patients were analyzed (Fig. 1). There were no significant differences in the patient characteristics and operation details between the two groups (Table 1). Intraoperative HR and MAP were comparable throughout the study period (Fig. 2).

**Table 1:** Patient’s characteristics and operation details.

	Control group (n = 32)	Patch group (n = 31)	P value
Age (years)	52 (42–63)	47 (40–61)	0.527
Height (cm)	158 (153–163)	159 (155–161)	0.581
Weight (kg)	61.3 ± 10.8	58.1 ± 9.8	0.229
BMI (kg/m^2^)	24 (22–27)	23 (21–25)	0.284
ASA physical status (1/2/3)	18/13/1	19/12/0	> 0.999
**Diagnosis**			0.743
Adenomyomatosis or polyps	9 (28%)	12 (39%)	
Cholecystitis			
Mild	12 (38%)	10 (32%)	
Moderate	2 (6%)	3 (10%)	
Severe	9 (28%)	6 (19%)	
Crystalloid (mL)	300 (275–400)	300 (275–400)	0.916
Bleeding (mL)	10 (10–20)	15 (5–20)	0.938
Total dose of remifentanil (μg)	400 (320–600)	350 (280–400)	0.055
Operation time (min)	50 (40–65)	50 (35–57.5)	0.229
Anesthesia time (min)	85 (70–97.5)	80 (65–90)	0.348

Values are presented as mean ± standard deviation, median (interquartile range) or number (proportion).

*BMI* body mass index, *ASA* American Society of Anesthesiologists.

The overall incidence of shoulder pain was significantly lower in the patch group than in the control group (42% vs. 78%, P = 0.005, Table 2). The incidence of shoulder pain at each time point except the baseline was also lower in the patch group. The number of patients showing more severe shoulder pain than abdominal pain was higher in the control group (P = 0.041), and the number of patients showing less shoulder pain compared to baseline was higher in the patch group (P = 0.024).

**Table 2:** Incidence of shoulder pain.

	Control group (n = 32)	Patch group (n = 31)	P value
**Incidence**^**a**^			
Overall	25 (78%)	13 (42%)	0.005
Baseline	4 (13%)	7 (23%)	0.337
30 min after surgery	6 (19%)	0	0.024
6 h after surgery	15 (47%)	6 (19%)	0.032
24 h after surgery	22 (69%)	11 (35%)	0.012
48 h after surgery	20 (63%)	8 (26%)	0.005
> Abdominal pain^b^	12 (37%)	4 (13%)	0.041
Alleviated pain^c^	0	5 (16%)	0.024

Values are presented as number (proportion).

^a^Incidence was defined as the number of patients having higher shoulder pain compared with baseline.

^b^The number of patients having worse shoulder pain compared with abdominal pain.

^c^The number of patients having less shoulder pain compared with baseline.

**Figure 3:**
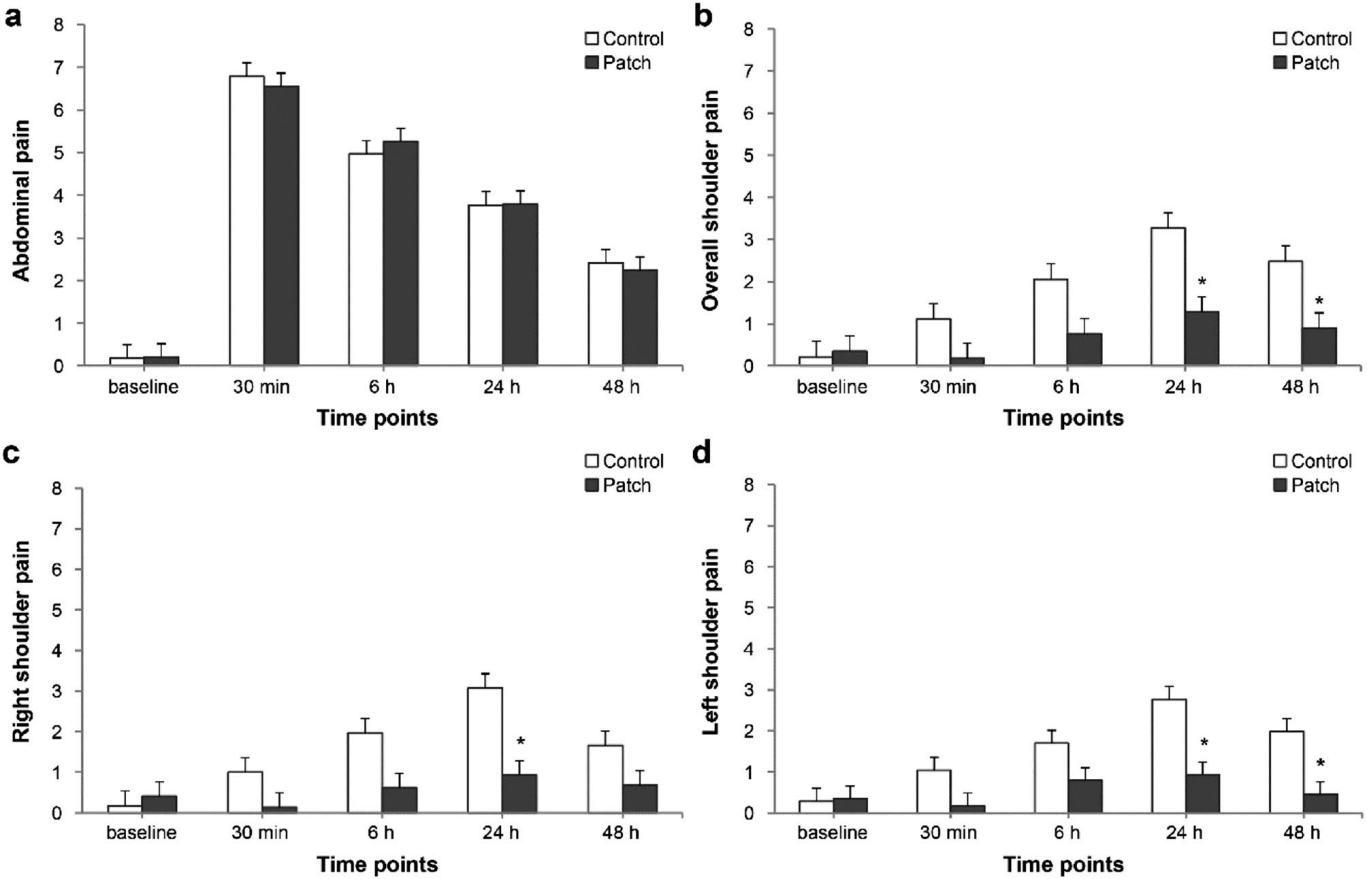
Changes of abdominal pain (**a**), and overall (**b**), right (**c**), and left shoulder pain (**d**) during the first 48 h after surgery. Values were expressed as mean ± standard error. *Baseline* before anesthesia induction, *30 min* 30 min after surgery, *6 h* 6 h after surgery, *24 h* 24 h after surgery, *48 h* 48 h after surgery. *P < 0.05 compared with the control group.

Abdominal pain showed a peak of severity at 30 min after surgery and gradually decreased thereafter in both groups (P_group*time_ = 0.868; Fig. 3a). Overall shoulder pain showed a peak of severity at 24 h after surgery in both groups (Fig. 3b). In addition, overall shoulder pain tended to be significantly different between the two groups over time (P_time_ < 0.001) and was significantly lower in the patch group than in the control group at 24 h and 48 h after surgery [mean value (SE); 1.3 (0.4) vs 3.3 (0.4), P_adjusted_ = 0.01 and 0.9 (0.4) vs 2.5 (0.4), P_adjusted_ = 0.015 at 24 h and 48 h, respectively]. Right shoulder pain was lower in the patch group at 24 h after surgery (P_adjusted_ = 0.01; Fig. 3c), and left shoulder pain was lower in the patch group at 24 h and 48 h after surgery (P_adjusted_ = 0.005 for both; Fig. 3d) compared with control group.

**Figure 4:**
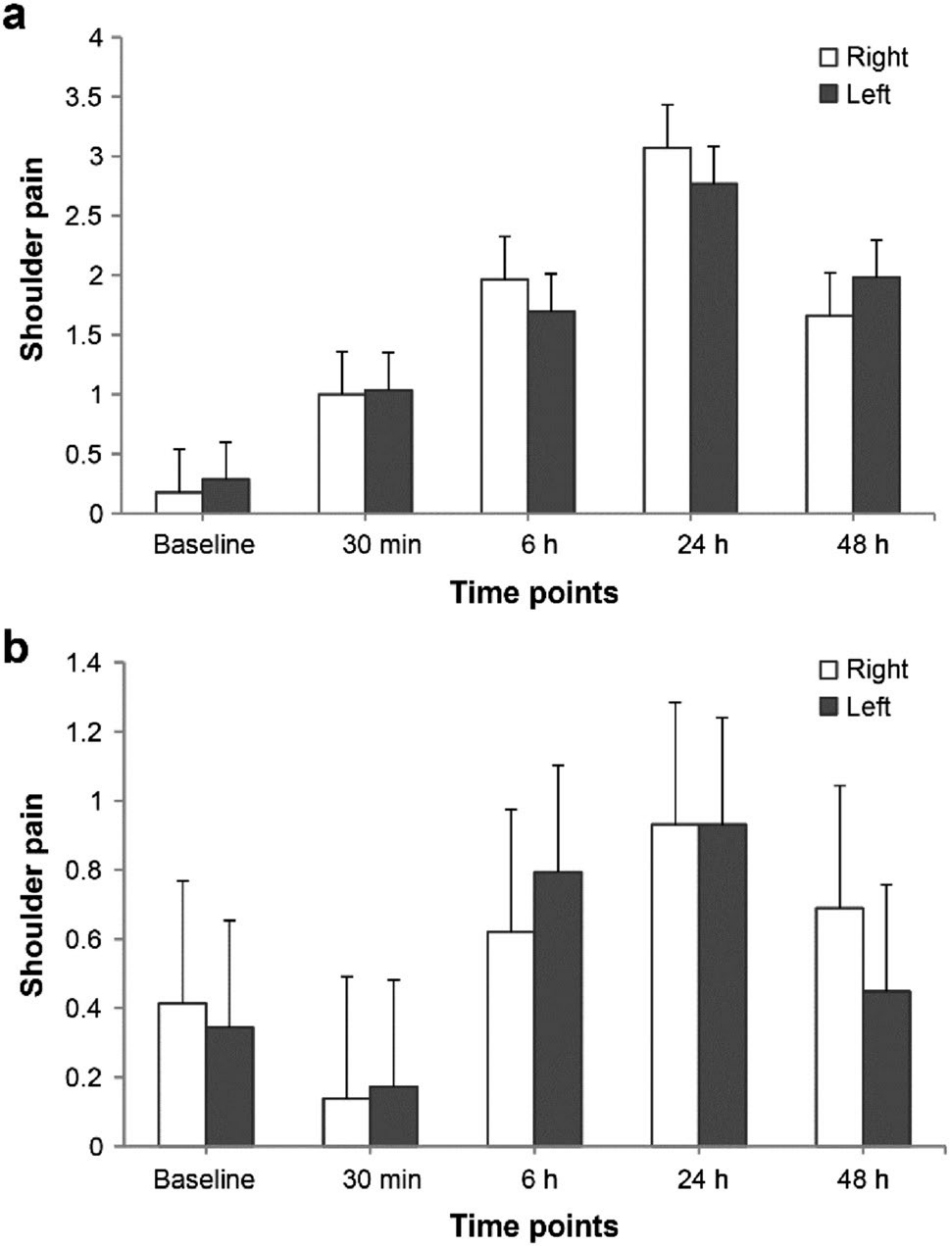
Comparison between right and left shoulder pain in control group (**a**) and patch group (**b**). Values were expressed as mean ± standard error. *Baseline* before anesthesia induction, *30 min* 30 min after surgery, *6 h* 6 h after surgery, *24 h* 24 h after surgery, *48 h* 48 h after surgery.

Right shoulder pain did not differ from left shoulder pain in either group (P_group*time_ = 0.613 and P_group*time_ = 0.449 in the control group and patch group, respectively; Fig. 4).

The recovery data were comparable between the two groups (Table 3). Nausea developed in 24 patients (12 patients in each group) during PACU or ward stay; no other complications related to the use of lidocaine patch 5% or dressing retention tape were found.

**Table 3:** Recovery profiles.

	Control group (n = 32)	Patch group (n = 31)	P value
**In PACU**			
Nausea	26/0/1/5	21/3/2/5	0.323
Vomiting	2 (6%)	2 (7%)	> 0.999
Patient requesting antiemetics	7 (22%)	8 (26%)	0.714
Patient requesting analgesic	25 (78%)	25 (81%)	0.805
Rescue fentanyl dose (μg)	57 (11–68)	54 (46–67)	0.803
Duration of PACU stay (min)	40 (30–50)	40 (40–50)	0.190
**At ward**			
Complications			
Fever	5 (16%)	3 (10%)	0.708
Urinary retention	2 (6%)	1 (3%)	> 0.999
Nausea	8 (25%)	4 (13%)	0.222
Vomiting	3 (9%)	2 (7%)	> 0.999
Hypotension	0	1 (3%)	0.492
Patient requesting antiemetics	2 (6%)	3 (10%)	0.672
Patient requesting analgesic	17 (53%)	19 (61%)	0.513
Hospital stay after surgery (day)	1 (1–2)	1 (1–1)	0.468

Values are presented as median (interquartile range) or number (%).

*PACU* post-anesthesia care unit.

## Discussion

This study demonstrated the beneficial analgesic effect of lidocaine patch 5% on decreasing shoulder pain after LC in female patients. The incidence of shoulder pain in the patch group was significantly reduced up to approximately 50% of that in the control group. The severity of shoulder pain also was significantly reduced in the patch group at 24 h and 48 h after surgery. The number of patients showing more severe shoulder pain than abdominal pain was higher in the control group, and the number of patients having less shoulder pain compared to baseline was higher in the patch group.

Although still unclear, the most probable mechanism of laparoscopy-related shoulder pain is the excitation of the phrenic nerve due to diaphragmatic or peritoneal irritation^2,3,6,19^. The phrenic nerve originates from the anterior branch of cervical spinal nerve roots C3–C5 and provides sensory innervation to the mediastinal pleura, pericardium, and peritoneal surfaces of the diaphragm^7,12^. The main nerve C4 also provides cutaneous innervation to the shoulder. Regarding the misinterpretation of the origin of input from the referred pain area^20,21^, diaphragmatic irritation during laparoscopy can provoke referred shoulder pain. Based on this “misinterpretation theory,” numerous strategies have been developed to reduce laparoscopy-related shoulder pain by minimizing diaphragmatic irritation. These interventions are sometimes effective, but the results are conflicting and there is no consensus on preventive measures.

A “pre-local hyper-excitability theory” has been proposed, in which stimuli in the initial area cause the hyper-excitation of the connective nerve between the referred area and initial area, consequently inducing the increased sensitivity of the referred area^22^. According to this theory, the primary pathogenesis is peripheral sensitization rather than central sensitization. In experimental studies on healthy volunteers, referred pain was partially decreased when the input from the peripheral receptors in the referred area was blunted, though conflicting results have been published^20,21^. For example, an EMLA cream over the referred skin area reduced the intensity of referred pain by 22.7%^23^, and a complete nerve block in the referred area reduced it by 40%^13^. In clinical studies on patients undergoing laparoscopic surgery, treatment on the shoulder effectively decreased referred shoulder pain after laparoscopy. For example, pretreatment using a trigger point injection or an EMLA cream on the shoulder significantly reduced the incidence and severity of shoulder pain after laparoscopy^14^. Moreover, transcutaneous electrical nerve stimulation on the shoulder alleviated shoulder pain during laparoscopy^24^. Based on these studies, we hypothesized that the application of a lidocaine patch to the shoulder could also reduce referred shoulder pain after laparoscopic surgery. In the present study, lidocaine patch 5% was applied to the referred pain area (the shoulder); consequently, the incidence and severity of shoulder pain after LC were reduced significantly.

Lidocaine patch 5% is a skin patch approved for the treatment of post-herpetic neuralgia. It is also used for localized and painful conditions such as vascular access, pain caused by trauma fracture, wound pain after surgery, and arthritis^18,25^. Each patch contains 700 mg of lidocaine in aqueous base, but only 2–3% of the dose is absorbed; the peak plasma level is 0.13 μg/mL (toxic level, 5 μg/mL), thus showing minimal adverse effects^26^. In a previous study, application of an EMLA cream on the shoulders reduced laparoscopy-related shoulder pain to an NRS score of < 1^14^, which was more effective than the lidocaine patch 5% used in present study (mean NRS scores of 1.3 and 0.9 at 24 h and 48 h after surgery, respectively). One of differences between the EMLA cream and the lidocaine patch is that EMLA produces local anesthesia by blocking large sensory fibers^15^ and the lidocaine patch exerts an analgesic effect by blocking the small sensory fibers without causing local anesthesia. Thus, the skin under the lidocaine patch has a normal sensation^15^. Despite the low analgesia potency, the lidocaine patch might be better for surgical patients than the EMLA cream due to the lack of numbness and occlusive dressing.

The peak shoulder pain score in this study was 1.3 at 24 h after surgery in the patch group. This was lower than the scores ranging from 1.9 to 4.2 in studies focusing on lessening diaphragmatic irritation during LC^12,17,27^. In addition, the present study only included female patients who have a lower pain threshold than male^28^. This is interesting finding that shoulder intervention showed more effective analgesia than diaphragmatic intervention during LC, because referred pain has been known to be mainly associated with central components (initial area) and not with peripheral components (referred area).

In the present study, shoulder pain after LC was reduced until 48 h after surgery despite the application of the lidocaine patch during the first 12 h. Lidocaine patch 5% has a half-life of 6–8 h^15^. In patients with myofascial pain syndrome, the effect of lidocaine patch 5% applied to three focal sites throughout the body for 4 days was superior to that of a placebo patch until day 9 after the beginning of treatment^16^. Similarly, in an area limited to the upper trapezius, a lidocaine patch applied for 7 days also relieved pain more effectively than a placebo patch for a period of 2 weeks^29^. There are two possible explanations for the long analgesic period of the lidocaine patch. First, after long-term application, lidocaine patch 5% decreases epidermal nerve fiber density without affecting pressure pain and threshold for heat- and cold-induced pain in the skin of healthy volunteers^30^. Second, central sensitization might play a role in persistent complaints in patients with shoulder pain^31^, although being poorly investigated. In the present study, the antinociceptive effect of lidocaine patch 5% that was initiated before the pneumoperitoneum might inhibit the central sensitization of the shoulder to some degree.

Right and left shoulder pain did not differ in the patch and control groups in present study. Shoulder pain after LC is generally more frequent in the right side^2^. During laparoscopic hysterectomy, right shoulder pain was more severe than left shoulder pain^32^. In contrast, Schoeffler et al. reported that more severe shoulder tip pain is noted in the left side in reference with protection of the right side of the diaphragm through the liver^33^. Further research is required to evaluate which side is more affected.

This study has several limitations. First, the sample size might be small when considering the simple intervention. Further studies are needed to verify our findings in a larger sample size. Second, shoulder pain scores were not evaluated by dividing separately during rest and movement. Third, when patients requested rescue analgesics, the main site of complaint was not evaluated. Fourth, more-than-mild pain (NRS ≥ 4) has considerable clinical significance. Regretfully, the number of patients with shoulder pain of NRS ≥ 4 was similar in this study (10 [31%] vs. 4 [13%] in the control vs. patch groups, P = 0.08). Fifth, at the time of patch removal by a nurse, the patient might have not remained blinded. Shame patches may be needed for complete blinding. Sixth, longer follow-up time of patients would be needed, because post-laparoscopic pneumoperitoneum was detected on upright chest radiographs in patients undergoing LC within the first week after surgery^34^.

In conclusion, lidocaine patch 5% reduced the incidence and severity of postoperative shoulder pain in female patients undergoing LC. Application of lidocaine patch 5% on the shoulder can be a simple, non-invasive, and effective analgesic method without adverse effects.

## Data Availability

The datasets generated during and/or analyzed during the current study are available from the corresponding author on reasonable request.
